# Macrophytes in Inland Waters: From Knowledge to Management

**DOI:** 10.3390/plants12030582

**Published:** 2023-01-29

**Authors:** Angelo Troia

**Affiliations:** Dipartimento di Scienze e Tecnologie Biologiche, Chimiche e Farmaceutiche (STeBiCeF), Università degli Studi di Palermo, 90123 Palermo, Italy; angelo.troia@unipa.it

**Keywords:** aquatic plants, hydrophytes, charophytes, wetlands, alien invasive species

## Abstract

The huge biodiversity of inland waters and the many different aquatic habitats or ecosystems occurring there are particularly threatened by human impacts. In this Special Issue, ten articles have been collected that show new data on the distribution and ecology of some rare aquatic macrophytes, including both vascular plants and charophytes, but also on the use of these organisms for the monitoring, management, and restoration of wetlands.

## 1. Introduction

The importance of freshwater on a global scale as a strategic element for the life of our species and for life in general is more clear today than ever before [1].

Inland waters [2], including not only freshwaters but also brackish or saline waters, host huge biodiversity and many different aquatic habitats or ecosystems that have unfortunately been increasingly threatened, disturbed, and damaged by human impacts in recent decades [1,3,4]. Indeed, we sometimes witness the destruction of important wetlands or aquatic biotopes before we are able to know their inhabitants (including their flora, fauna, microbiota, etc.).

Focusing on the “green” component, the strictly aquatic flora of inland waters (i.e., the plants living in the water or on its surface) are generally poorly known, probably because most “classic” botanists often stop at the edge of the land/water border. In addition, the evolutionarily and ecologically key group of charophytes is often not studied because phycologists focus on marine species (and charophytes usually occur only in inland waters) while botanists think that charophytes are not “plants” but just “algae”, so outside their skills or kingdom.

The aim of this Special Issue was to invite people studying aquatic plants (and charophytes) in inland waters to contribute to furthering the knowledge on these organisms, with basic (taxonomical or ecological) or applied research (involving the management of aquatic plants, or their use in assessing the quality of waters or the quality of environments), with a special focus on floating and submersed macrophytes (i.e., the so-called hydrophytes). Invasive alien aquatic species, of course, were also possible subjects.

## 2. Special Issue Contents

As mentioned in the introduction, aquatic macrophytes are often underknown, so even reports on new populations are often interesting to better understand, for example, the biology, biogeography, or ecology of species. Sciandrello et al. [5] report a new population of the “marsh fern” *Thelypteris palustris* in the well-known island of Sicily, at the southern border of its distribution area. Trbojević et al. [6] report a new population of *Chara baueri* in Serbia: this is an important record, since the species is very rare, with few known populations in Europe and one in Asia. In both articles, the authors supply significant information on the morphology and ecology of the new and isolated populations.

A different case is the report on *Chara zeylanica* in Sardinia by Becker et al. [7]: the new population, in fact, is not only the first one in Europe, but—according to the scenario presented by the authors—also a possible case of “range-shifting”; in other words, the species could be shifting its distribution (in this case northwards), probably due to climate change [8].

Turner et al. [9] focused on *Stratiotes aloides* L., a vascular macrophyte with a wide distribution (from northern Central Europe in the west to Siberia in the east) but that is becoming rare in some areas; in their article, they show the importance of assessing the genetic structure of a species in order to manage it, both for preserving the diversity of the species as a whole and for possible reintroductions. As in other species, the authors verified that, in European populations of this aquatic plant, there is low genetic diversity within each population but high genetic diversity between populations.

Millozza and Abdelahad [10] present a different aspect of the basic research, more connected to the taxonomy. In detail, this paper provides an example of how historical herbarium collections can be used to assist ecology, biogeography, and conservation biology research, in this case supplying information to better define a species described at the end of the nineteenth century.

The articles of Peternel et al. [11] and Panzeca et al. [12] present two different examples in which macrophytes are used to characterize aquatic environments, in a river in Slovenia and in farm ponds of a district of Sicily, respectively. The species composition of the macrophyte community revealed significant changes over the years in a riverine ecosystem in the first case, whereas in the second one it was shown that, although farm ponds are artificial and relatively poor habitats, they seem to be important for aquatic flora and the conservation of local biodiversity.

The article by Ribaudo et al. [13] introduces more proper management aspects. This paper is the only one (in this Special Issue) dealing with alien invasive species, one of the main threats today to the conservation of species and ecosystems on a global scale, but its approach is original and invasive species are not even mentioned in the title or the abstract. The study aims at linking the role of wind action and water oxygenation within dense hydrophyte stands in two shallow lakes located in the southern Atlantic coast of France. Its results highlight the need to consider local hydrodynamics in lake management decisions, and show that mapping hypoxia risk in densely vegetated stands is a promising tool for the management of invasive hydrophytes in shallow lakes. Furthermore, the two invasive alien aquatic species are two submerged rooted Hydrocharitaceae, *Egeria densa* Planch. and *Lagarosiphon major* (Ridl.) Moss.

The re-establishment of submerged macrophytes, and especially charophyte vegetation, is a common aim in wetland management. The contribution of Blindow et al. [14] reviews the knowledge on the life forms, dispersal, establishment, and transplantations of submerged macrophytes, focusing on charophytes, and provides recommendations for an ambitious Swedish project that aims to protect threatened macrophyte species. Rodrigo [15] reviews the available knowledge in wetland restoration based on revegetation with hydrophytes and stresses common challenges as well as potential solutions; the clear negative factors which prevent revegetation success are considered, and useful final suggestions are provided.
